# Using Small RNA Deep Sequencing Data to Detect Human Viruses

**DOI:** 10.1155/2016/2596782

**Published:** 2016-03-15

**Authors:** Fang Wang, Yu Sun, Jishou Ruan, Rui Chen, Xin Chen, Chengjie Chen, Jan F. Kreuze, ZhangJun Fei, Xiao Zhu, Shan Gao

**Affiliations:** ^1^Department of Gynaecology, The Second Hospital, Tianjin Medical University, Tianjin 300211, China; ^2^College of Life Sciences, Nankai University, Tianjin 300071, China; ^3^School of Mathematical Sciences, Nankai University, Tianjin 300071, China; ^4^Tianjin Institute of Agricultural Quality Standard and Testing Technology, Tianjin Academy of Agricultural Sciences, Tianjin 300381, China; ^5^College of Horticulture, South China Agricultural University, Guangzhou, Guangdong 510642, China; ^6^International Potato Center (CIP), Apartado 1558, Lima 12, Peru; ^7^Boyce Thompson Institute for Plant Research, Cornell University, Ithaca, NY 14853, USA; ^8^Guangdong Provincial Key Laboratory of Medical Molecular Diagnostics, Dongguan Scientific Research Center, Guangdong Medical University, Dongguan, Guangdong 523808, China

## Abstract

Small RNA sequencing (sRNA-seq) can be used to detect viruses in infected hosts without the necessity to have any prior knowledge or specialized sample preparation. The sRNA-seq method was initially used for viral detection and identification in plants and then in invertebrates and fungi. However, it is still controversial to use sRNA-seq in the detection of mammalian or human viruses. In this study, we used 931 sRNA-seq runs of data from the NCBI SRA database to detect and identify viruses in human cells or tissues, particularly from some clinical samples. Six viruses including HPV-18, HBV, HCV, HIV-1, SMRV, and EBV were detected from 36 runs of data. Four viruses were consistent with the annotations from the previous studies. HIV-1 was found in clinical samples without the HIV-positive reports, and SMRV was found in Diffuse Large B-Cell Lymphoma cells for the first time. In conclusion, these results suggest the sRNA-seq can be used to detect viruses in mammals and humans.

## 1. Introduction

Infection by pathogens is one of the main risk factors for many diseases [1–4], particularly for cancers. In 2008, approximately two million new cancer cases (16%) worldwide were caused by pathogen infection. Most cancers inducing infectious agents were viruses [5], including Epstein-Barr virus (EBV), hepatitis B and C virus (HBV and HCV, resp.), Kaposi sarcoma herpes virus (KSHV, also known as human herpes virus type 8, HHV-8), human immunodeficiency virus type 1 (HIV-1), human papillomavirus type 16 (HPV-16), and human T-cell lymphotropic virus type 1 (HTLV-1). Therefore, the rapid and accurate detection and identification of these viruses is essential to human health. Conventional detection methods (e.g., ELISA, PCR, or microarrays) cannot be used in some cases due to failure to satisfy certain requirements (e.g., prior knowledge of the potential pathogen or the ability to cultivate and purify the pathogen [6]). In addition, they are time-consuming and difficult to use in detection of highly divergent or novel viruses.

To overcome these limitations, next generation sequencing (NGS) technologies have been applied for virus and viroid discovery in plants and animals [7, 8]. Compared to other NGS based methods requiring the use of viral enrichment and concentration procedures [7], the small RNA sequencing (sRNA-seq) based method simplifies the virus detection, with the aid of virus fragments enriched by the RNA interference (RNAi) mechanism. RNAi is a cytoplasmic cell surveillance system which recognizes double-stranded RNA (dsRNA) and specifically destroys single-stranded RNA and dsRNA molecules homologous to the dsRNA inducer, using small interfering RNAs (siRNAs) as a guide [9]. The abundant siRNAs accumulated during the RNAi process facilitate virus detection and the study of RNAi mechanism. RNAi has been proposed as a key antiviral intrinsic immune response in plants, nematodes, and arthropods [10]. Based on such theory, the sRNA-seq method was originally used for viral detection and identification in plants [8, 11, 12] and in invertebrates [13–15], but not in mammals or humans. There was evidence that antiviral RNAi functions in mammalian germ cells and embryonic stem cells (ESCs), as well as some carcinoma cell lines [10]. No evidence had been provided to prove RNAi functions in mammalian somatic cells until Li et al.'s work was published [16]. Although Li et al. discovered low level siRNA duplexes in the baby hamster kidney 21 cells, the role of RNAi in viral defence in mammalians remains controversial. Therefore, using sRNA-seq to detect viruses in mammals and humans is a highly promising but hard topic.

In this study, we used 931 sRNA-seq runs of data from the NCBI SRA database [17] to detect and identify viruses in human cells or tissues, particularly from some clinical samples. These tissues came from saliva, tongue, laryngopharynx, oropharynx, prefrontal cortex, liver, cervix, serum, plasma, lymph, and so forth. As a result, six viruses including HPV-18, HBV, HCV, HIV-1, SMRV (squirrel monkey retrovirus), and EBV were detected from 36 runs of data. In brief, the existence of HPV-18, HBV, HCV, and EBV was consistent with the findings from the original studies, whereas HIV-1 and SMRV had not been identified previously in the experimental samples. The nucleotide polymorphism, read-enriched regions (hotspots), and RNAi responses of detected viruses were analyzed, following the detection of these viruses.

## 2. Materials and Methods

Using NCBI SRA advanced searching tools (http://www.ncbi.nlm.nih.gov/sra/advanced), we retrieved 2,820 runs of data by the combined keywords including Illumina, small RNA, and* Homo sapiens* (November 1, 2014). We subsequently filtered these data based on the following criteria: (1) to remove non-small RNA-seq data by reading the annotations; (2) to remove data containing keyword “cell line”; (3) to remove data from cDNA library selection during library construction. Ultimately, we used 931 runs of data from 42 previous studies in this study (Table 1).

The software Fastq_clean [18] was used for sRNA data cleaning and quality control. To detect and identify viruses using sRNA-seq data, we developed an automatic pipeline using Perl scripts. This pipeline had performed well in the detection and identification of plant and insect viruses in our previous studies [12, 19–21]. The pipeline integrated three sequence databases: The first one was an rRNA database, which was built based on the SILVA ribosomal RNA gene database [22]. The second one was the human host genome for the subtraction of host genome sequences. The last one contained the Vertebrata viral sequences constructed from the NCBI GenBank database, version 197. The relationship information between the virus genus and the host was from the International Committee on Taxonomy of Viruses (ICTV). For some virus genera which did not have host information assigned to them, we were able to assign host categories after reading their NCBI annotations.

For each detected virus, we assigned a putative reference genome from the NCBI GenBank database to represent the virus (Supplementary File 1 in Supplementary Material available online at http://dx.doi.org/10.1155/2016/2596782). We used the reference genome coverage and the average depth to quantify the detected viruses. The genome coverage represents the proportion of read-covered positions against the genome length. The average depth is equal to the total base pairs of the aligned reads divided by the read-covered positions on the reference genome (Tables 2 and 3).

## 3. Results and Discussion

### 3.1. HPV-18 from HeLa Cell Lines

To test our virus detection pipeline, we used HeLa cell line data from the previous study SRP001381 as positive controls (Table 1). The HeLa cell line, derived from cervical cancer cells of the patient Henrietta Lacks, contains HPV-18, one of the carcinogenic HPV genotypes. In this study, HPV-18 was detected in all of the three runs of data (SRR031635, SRR031636, and SRR031637). The assembled HPV-18 in the data SRR031636 covered 74.1% of the reference genome M20325 with an average depth 8.5. The 19 long viral contigs (≥ 40 bp) covered 62.5% of the reference genome with a uniform distribution (Supplementary File 1).

### 3.2. HBV and HCV from Human Liver and HCC Tissues

Chronic hepatitis B virus (HBV) is one of the first viruses to be causally linked to a human tumor and is a major global cause of hepatocellular carcinoma (HCC). HBV, hepatitis C virus (HCV), and cirrhosis between them contribute to the genesis of almost all global HCCs [23]. Conventional clinical tests use markers at the protein level, including the HBV surface antigen (HBsAg), HBV envelope antigen (HBeAg), and HBV core antigen (HBcAg) and their antibodies from the patients' serum. However, these protein markers are not always present for various reasons [23].

In the previous study SRP002272 from the NCBI SRA database (Table 2), 15 clinical samples had been sequenced including three normal liver tissues, one HBV-infected liver tissue, one severe chronic hepatitis B liver tissue, two HBV-positive HCC tissues, one HCV-positive HCC tissue, and one HCC tissue without HBV or HCV [24]. In this study, the detection and identification results in 15 runs of data were consistent with the findings from the previous study SRP002272 with one exception SRR039619 (Table 2). The sRNA data SRR039619 from a HBV-positive HCC patient should have contained HBV but it was not found by our pipeline. SRR039619 contained 9,161,157 reads, which possibly were not deep enough to catch adequate virus derived small RNAs (vsRNAs) for detection.

The assembled HBV in the data SRR039620 covered 54.6% of the reference genome JQ688404 with an average depth 6 (Supplementary File 1). In the data SRR039620, seven long viral contigs (≥ 40 bp) covered the HBV x (HBx), HBV core (HBc), and HBV polymerase (HBp) gene regions but did not cover the HBV surface (HBs) gene region. The long viral contigs (≥ 40 bp) in the data SRR039614 and SRR039621 only covered the HBx gene region. The assembled HCV in the data SRR039622 covered 10.8% of the reference genome D85516 with an average depth 1 (Supplementary File 1). HCV was also detected in the data SRR039623 with genome coverage 8.3% and average depth 1.

### 3.3. HIV-1 from Breast Cancer Patients

HIV as a member of the genus* Lentivirus* causes acquired immunodeficiency syndrome (AIDS). As technology evolves, HIV testing assays are being improved on sensitivity and specificity [25]. However, the tests still provide false negative results due to the diagnostic window or other reasons [25]. In the previous study SRP027589 from the NCBI SRA database (Table 1), 42 samples had been sequenced for the discovery and profiling of circulating microRNAs in the serum of 42 stage II-III locally advanced and inflammatory breast cancer (BC) patients [26]. These patients received neoadjuvant chemotherapy (NCT) followed by surgical tumor resection. However, no AIDS or HIV-positive results of these patients had been reported in the previous study SRP027589. In this study, HIV-1 was detected at a very high level in the data SRR941591. The assembled HIV-1 in the data SRR941591 covered 39.3% of the reference genome M19921 with an average depth 210.1 (Supplementary File 1). As far as we know, this was the first time to report the detection of HIV-1 using sRNA data from clinical samples.

### 3.4. SMRV and EBV from B Cells and Exosomes

SMRV, an endogenous virus of squirrel monkeys, had been isolated by cocultivation of squirrel monkey lung cells with canine cells [27]. In previous studies, SMRV had been detected in Burkitt's lymphoma (BL) cell lines [28]. Specifically, the insertion of the incomplete SMRV proviral genomes had been detected in Namalwa cell lines [29]. However, we found no reports that SMRV had been detected in the Diffuse Large B-Cell Lymphoma (DLBCL). To the best of our knowledge, DLBCL had only been reported to be caused by EBV [30], HCV [31], HIV [32], and SV40 (Simian Virus 40) [33].

In this study, SMRV was detected in the data SRR1563015 and SRR1563017 (Table 3). The assembled SMRV in these two runs of data covered 99.2% and 99.4% of the reference genome M23385 at an average depth of 146.1 and 494.5, respectively. In the data SRR1563017, the longest viral contig was assembled to have a length of 6,760 bp and an identity 99% (6,751/6,764) of the reference sequence M23385 (Supplementary File 1). As far as we know, this was the first time to report the detection of SMRV using sRNA data from DLBCL samples.

Epstein-Barr virus (EBV) has been firmly linked to some cancers and proliferative diseases, including Burkitt's lymphoma (BL), nasopharyngeal carcinoma, immunoblastic lymphoma, a subset of gastric carcinomas, rare T- and NK-cell lymphomas or leiomyosarcoma, acute infectious mononucleosis, and Hodgkin's disease. Almost 100% of BL cases in Equatorial Africa carry EBV. Children infected early in life with the highest antibody titres to the virus are at the highest risk of developing the tumor [34]. EBV-positive BL predominant in Africa and EBV-negative BL predominant in Europe and/or the United States have different causation and characteristics [34].

In the previous study SRP046046 from the NCBI SRA database, 12 samples had been sequenced to distinguish the small RNA composition in six B cells from their exosomes. Six B cells included three EBV-positive lymphoblastoid B cells (LCLs), one EBV-positive Burkitt's lymphoma (BL) cell, one EBV-negative BL cell, and one Diffuse Large B-Cell Lymphoma (DLBCL) cell. As a result, EBV had been detected from two EBV-positive BL samples and six EBV-positive LCL samples (Table 3). In this study, EBV was detected in the data SRR1563018, SRR1563056, SRR1563059, SRR1563060, SRR1563061, SRR1563062, SRR1563063, and SRR1563064. This finding confirmed the results in the previous study SRP046046. However, the reference genome coverage by vsRNAs was uneven in eight runs of data varying from 1.6% to 31.4%. This large variance could result from sample extraction, small RNA library construction, sequencing quality, or sequencing depth. In the data SRR1563063, the assembled EBV contigs covered 20.6% of the reference genome M80517 (Supplementary File 1).

### 3.5. Nucleotide Polymorphism, Hotspots, and RNAi Responses

**Figure 1:**
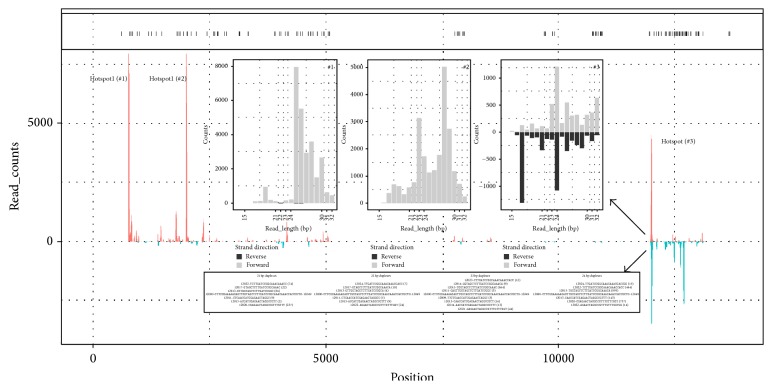
Nucleotide polymorphism, hotspots, and siRNA duplexes of HIV-1. The *x*-axis represents positions on the HIV-1 reference genome (GenBank: M19921). The *y*-axis represents the read counts from the data SRR941591 on each position. The dots in the top black box represent positions with polymorphic nucleotides. #1, #2, and #3 are the size distributions of positive- and negative-strand viral reads in hotspot 1 (779–810 bp), hotspot 2 (2,017–2,045 bp), and hotspot 3 (12,006–12,044 bp). The read counts of 21 bp, 22 bp, 23 bp, and 24 bp siRNA duplexes are marked in parentheses.

The plant sRNA-seq data had been shown to contain adequate information for studying nucleotide polymorphism of the actual virus [35]. Among the six human viruses found in this study, HIV-1 in the data SRR941591 showed the highest nucleotide polymorphism rate covering 2.66% (155/5,831) of the genomic positions (Figure 1), as compared to SMRV in the data SRR1563017, EBV in the data SRR1563063, and HPV-18 in the data SRR031636 covering only 0.41% (36/8,732), 0.29% (110/37,898), and 0.13% (3/2,324) of the genomic positions, respectively (Supplementary File 2). HIV-1 is a single-stranded RNA (ssRNA) reverse-transcribing virus. HIV reverse transcriptase has been shown to be exceptionally inaccurate [36] and may explain the high polymorphism rates observed in this study. HPV-18 and EBV are double-stranded DNA (dsDNA) viruses which have low error rates during their replication. SMRV, as ssRNA retrovirus, was expected to have a high nucleotide polymorphism rate but this was not reflected in these data. HBV and HCV showed no polymorphism whatsoever, probably due to the low sequencing depth.

Consistent with our previous results in plant virus detection, the distribution of vsRNA coverage over the human virus genomes was not even, with some read-enriched regions (hotspots) in the vsRNA-covered regions on both of the positive and negative strands (Supplementary File 3). In HPV-18, HBV, HCV, and SMRV, the vsRNA-covered region on the positive strand was more than nine times larger than the vsRNA-covered region on the negative strand, while HIV-1 and EBV had little difference between vsRNA-covered regions over the positive and negative strands. Using the data SRR941591 as an example (Figure 1), the number of bases covered by vsRNA reads on the HIV-1 positive strand against the negative strand was 4,961 bp to 3,945 bp with overlap 52.74% (3,075/5,831). There were three obvious hotspots on the putative HIV-1 reference M19921. The first (779–810 bp) and second (2,017–2,045 bp) hotspot resided on the HIV-1 positive strand. Different from the first and second hotspot, the third hotspot (12,006–12,044 bp) consisted of positive- and negative-strand vsRNAs.

**Figure 2:**
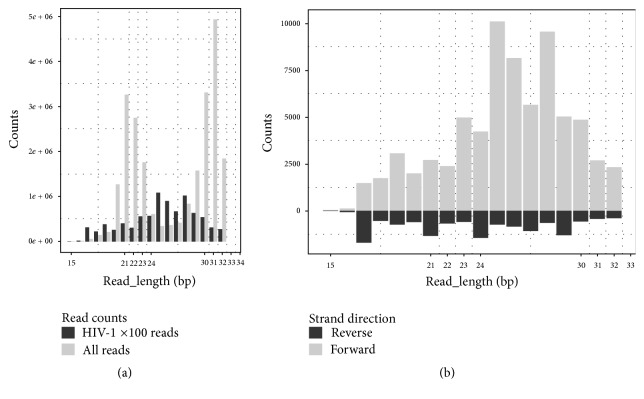
Distribution of the total and HIV-1 viral read length on both of the strands. The *x*-axis represents read length. The *y*-axis represents the read counts of each length in the data SRR941591. HIV-1 ×100 reads represent 100 times of reads which can be aligned to the HIV-1 reference genome (GenBank: M19921).

To investigate the RNAi responses using 36 virus-containing runs of data, we analyzed the length distribution of the reads aligned to the virus reference sequences. Viral small RNA read lengths of HIV-1 in the data SRR941591 had the distribution pattern expected from a RNAi response, similar to what had been found in previous studies [16]. This pattern consists of positive- and negative-strand vsRNAs with countable values at the 21, 22, 23, and 24 bp read length (Figure 2). Another characteristic of RNAi responses is that there must be positive- and negative-strand vsRNAs in some hotspots. In the data SRR941591, the third hotspot satisfied this criterion. The last and key step to identify RNAi responses is to find the siRNA duplexes from hotspots. They are usually only a minute fraction of the total vsRNAs, because the duplexes are short lived, due to one of the two strands being rapidly degraded following their creation. In the third hotspot, we found three canonical 22 bp siRNA duplexes containing a 20 nt perfectly base-paired duplex region with 2 nt 3′ overhangs. We also found 21, 23, and 24 bp siRNA-like duplexes, respectively (Figure 1). However, we used the putative HIV-1 reference M19921 for this analysis, without knowledge of the exact HIV-1 sequence. M19921 is recombinant clone pNL4-3, which includes the HIV-1 virus region and vector region. Since the pNL4-3 clone is only constructed for the experiment use, the vsRNAs on the pNL4-3 vector region could be contamination during the library construction or sequencing process. Although the third hotspot existed in the pNL4-3 vector region, rather than the HIV-1 region on the reference M19921, the reliability of these siRNA duplexes in the third hotspot was supported by their uniqueness in the NCBI GenBank database and high sequencing depth. Therefore, this RNAi response could have happened in other samples.

**Figure 3:**
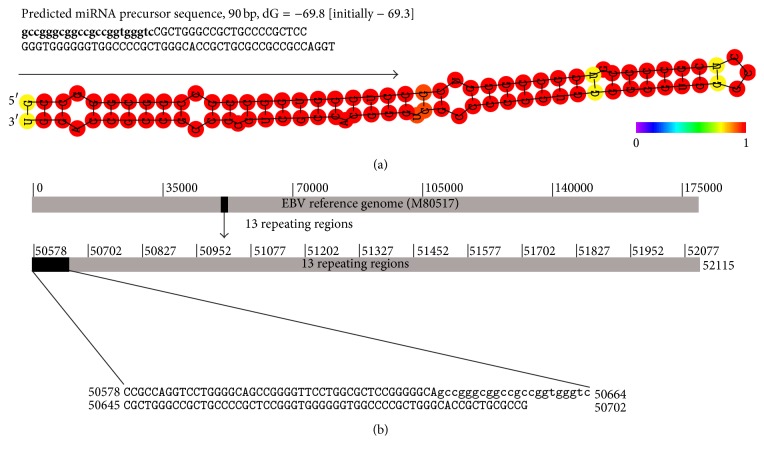
The predicted miRNAs of EBV. The EBV detected in data SRR1563063 is represented using the reference genome (GenBank: M80517) in this study. The sequence of the predicted mature miRNA is represented using the lowercase letters. (a) The second structures of the miRNA were predicted using RNAfold. (b) The first repeating unit (50578-50702) contains the predicted mature miRNA (50624-50646). This mature miRNA is repeated 12 times in 13 repeated units.

In addition to the siRNAs, vsRNAs include other small RNA reads, for example, microRNAs (miRNAs), piwi-interacting RNAs (piRNAs), or degraded mRNA fragments. DNA viruses produce their own miRNAs facilitating the detection of DNA viruses, for example, EBV. In the data SRR1563063, we found seven miRNA-like duplexes from EBV vsRNAs (Supplementary File 3). Then, we blasted these duplexes to the miRBase database (http://www.mirbase.org/) and identified three known mature virus miRNAs. They were rlcv-mir-rL1-1-3p, ebv-mir-BART1-5p, and ebv-mir-BART1-3p located at positions 53,801, 151,640, and 151,676 on the EBV reference M80517. Three mature miRNAs came from two miRNA precursors (pre-miRNAs) rlcv-mir-rL1-1 and ebv-mir-BART1. Compared to two other miRNAs, the ebv-mir-BART1-5p was expressed at a very high level of 17,987 read counts. As for the remaining four duplexes, we confirmed they could not be matched to the human genome. Then, we used RNAfold online server (http://rna.tbi.univie.ac.at/cgi-bin/RNAfold.cgi) to predict the second structures of their pre-miRNAs (Supplementary File 4). As a result, we identified a new EBV pre-miRNA, with a length of 90 bp and a very high minimal folding free energy index (MEFI) over 1.0 (Figure 3(a)). Furthermore, this pre-miRNA resided in a repeating region on the reference M80517. This repeating region from 50,578 to 52,077 bp had 13 units (Figure 3(b)). Each unit with a length of 125 bp contained this pre-miRNA sequence. It suggested that this repeating region comprise a primary miRNA (pri-miRNA).

## 4. Conclusions

In this study, we used 931 sRNA-seq runs of data from the NCBI SRA database to detect and identify human viruses. Six viruses were detected and two of them were not found in previous studies. These results suggest the sRNA-seq can be used to detect viruses in mammals and humans. The sRNA-seq data contains the heterozygosity information that can be used to investigate the pathogen evolution in one person and design therapies to deal with a specific virus population. The sRNA-seq data can also be used to find new virus miRNA or to investigate the RNAi responses in mammals and humans. However, sRNA-seq data used in this study were not from virus-infection experiments with knowledge of the exact virus sequences. In place of the exact virus sequences, the putative virus sequences were used to investigate the RNAi responses. Although using the putative virus sequences brought some uncertainties, the results of this study still shed light on the studies of virus induced RNAi in mammals.

## Supplementary Material

Supplementary Material includes four files, which are Detection reports of six viruses, Read distribution on six virus genomes, The sequences and loci of seven duplexes, and The predicted second structures of seven duplexes.

## Figures and Tables

**Table 1:** The 42 previous studies from the SRA database.

Study ID	Runs	Sample source	Disease
DRP000998	3	Whole saliva, salivary exosome	Healthy
ERP001908	63	Tongue, laryngopharynx, oropharynx	HNSCC
ERP004592	23	Prefrontal cortex	Huntington's disease
SRP001381	3	HeLa cell line	HPV18(+)
SRP002118	14	Hek293T cell line	NA
SRP002272	15	Liver	HBV(+), HCV(+), HCC
SRP002326	38	Cervical tumor	Cervical cancer
SRP002402	3	Sperm	Healthy
SRP007825	67	Skin	Psoriasis
SRP008258	2	Hek293, HeLa cell line	NA
SRP009246	4	Primary human fibroblast	NA
SRP014020	20	Thyroid tumor	Follicular thyroid adenoma
SRP017809	4	Dorsolateral prefrontal cortex	Healthy
SRP017979	4	Colorectal tumor	Colorectal cancer
SRP018255	35	Plasma, serum, placenta	Healthy
SRP021130	20	Cerebral cortex	FTLD, PSP, BHS, DLB, Alzheimer's disease
SRP021193	40	Heart	NIC, IC
SRP021911	12	Cumulus granulosa cell, mural granulosa cell	NA
SRP021924	5	Brain frontal cortex	NA
SRP022043	70	Blood	Alzheimer's disease
SRP022054	26	Sigma, liver, coecum, colon ascendens, lymph node	Colorectal cancer
SRP026081	2	Penicillium marneffei	NA
SRP026558	2	PBMC	Osteopetrosis
SRP026562	11	Prefrontal cortex	Alzheimer's disease
SRP027589	42	Serum	Breast cancer
SRP028291	78	ACA, ACC tumor, adrenal tissue	ACA, ACC
SRP028738	16	MiRQC, serum, liver	NA
SRP029599	9	FFPE, serum	Nonkeratinizing NPC, NPC
SRP032650	4	Serum	Latent PTB, PTB
SRP032953	12	Alpha cell, beta cell, whole islet	Type 2 diabetes mellitus
SRP033505	3	Plasma	Healthy
SRP033566	185	Connective tissue, plasma, neuronal tissue, primary cell, cardiac muscle, epithelium, skeletal muscle	DCM, IC
SRP034547	4	Primary fibroblast	Microcephaly
SRP034586	24	Serum, PBMC	Healthy
SRP034590	14	Plasma	NA
SRP034654	12	Tensor fascia lata, quadricep vastus, vastus externe, rhomboid, iliopsoas	FSHD
SRP034698	8	Skin, lymph node	MCC, SCC, melanoma, BCC
SRP040421	12	Exosome in human semen	Healthy
SRP041082	2	Seminal fluid	Prostate cancer
SRP046046	12	Lymphoblastoid	DLBCL, Burkitt's lymphoma, EBV(+)
SRP046234	2	Breast epithelium	Triple negative breast cancer
SRP048290	6	Platelet	Healthy

“Study ID” is uniq for each high-throughput project in the NCBI SRA database. ACA: adrenal cortical adenoma, ACC: adrenal cortical carcinoma, BCC: Basal Cell Carcinoma, BHS: bilateral hippocampal sclerosis, DCM: Dilated Cardiomyopathy, DLB: dementia with Lewy bodies, DLBCL: Diffuse Large B-Cell Lymphoma, FSHD: Facioscapulohumeral Muscular Dystrophy, FTLD: frontotemporal lobar dementia, HCC: HBV-related hepatocellular carcinoma, HNSCC: Head and Neck Squamous Cell Carcinoma, IC: Ischemic Cardiomyopathy, MCC: Merkel Cell Carcinoma, NIC: Nonischemic Cardiomyopathy, NPC: nasopharyngeal carcinoma, PBMC: Peripheral Blood Mononuclear Cell, PSP: Progressive Supranuclear Palsy, PTB: Pulmonary Tuberculosis, and SCC: Squamous Cell Carcinoma.

**Table 2:** HBV and HCV from the SRP002272 study.

Run ID	Sample_Source	Reference	Cov (%)	Depth
SRR039611	Human Normal Liver Tissue	NA	NA	NA
SRR039612	Human Normal Liver Tissue	NA	NA	NA
SRR039613	Human Normal Liver Tissue	NA	NA	NA
SRR039614	HBV-Infected Liver Tissue	JQ688405	423 (13.2)	3.0
SRR039615	Severe Chronic Hepatitis B Liver Tissue	NA	NA	NA
SRR039616	HBV(+) Distal Tissue	NA	NA	NA
SRR039617	HBV(+) Adjacent Tissue	NA	NA	NA
SRR039618	HBV(+) Side Tissue	NA	NA	NA
SRR039619^*∗*^	HBV(+) HCC Tissue	NA	NA	NA
SRR039620	HBV(+) Adjacent Tissue	JQ688404	1756 (54.6)	6.0
SRR039621	HBV(+) HCC Tissue	GQ475344	321 (10)	1.5
SRR039622	HCV(+) Adjacent Tissue	D85516	1032 (10.8)	1.8
SRR039623	HCV(+) HCC Tissue	GU133617	805 (8.3)	8.0
SRR039624	HBV(−) HCV(−) Adjacent Tissue	NA	NA	NA
SRR039625	HBV(−) HCV(−) HCC Tissue	NA	NA	NA

“Run ID” is uniq for each high-throughput fastq file in the NCBI SRA database. “Reference” uses the NCBI GenBank accession number. “Cov (%)” and “Depth” represent the genome coverage and the average depth, respectively. “Side Tissue” is close to the border between the tumor tissues and the normal tissues but 0–2 cm far from the tumor tissues. “Adjacent Tissue” is the normal tissues 2–5 cm far from the tumor tissues. “Distal Tissue” is the normal tissues at least 10** **cm far from the tumor tissues. “SRR039619^*∗*^” should have contained HBV but it was not found by our pipeline.

**Table 3:** SMRV and EBV from the SRP046046 study.

Run ID	Sample_Source	Reference	Cov (%)	Depth
SRR1563015	DLBCL	M23385	8714 (99.2)	146.1
SRR1563017	DLBCL Exosome	M23385	8732 (99.4)	494.5
SRR1563018	EBV(+) BL	KC207813	2765 (1.6)	29.2
SRR1563056	EBV(+) BL Exosome	KC207813	33107 (19.3)	9.6
SRR1563057	EBV(−) BL	NA	NA	NA
SRR1563058	EBV(−) BL Exosome	NA	NA	NA
SRR1563059	EBV(+) LCL	KC207813	13757 (8)	358.2
SRR1563060	EBV(+) LCL Exosome	M80517	7444 (4)	288.8
SRR1563061	EBV(+) LCL	M80517	18688 (10.2)	151.1
SRR1563062	EBV(+) LCL Exosome	KC207814	7931 (4.6)	198.2
SRR1563063	EBV(+) LCL	M80517	37898 (20.6)	52.8
SRR1563064	EBV(+) LCL Exosome	M80517	57850 (31.4)	17.6

“Run ID” is uniq for each high-throughput fastq file in the NCBI SRA database. “Reference” uses the NCBI GenBank accession number. “Cov (%)” and “Depth” represent the genome coverage and the average depth, respectively.
