# Supplementary material for: Using Small RNA Deep Sequencing Data to Detect Human Viruses
Source: Biomed Res Int. 2016 Mar 15;2016:2596782. doi: 10.1155/2016/2596782 (PMC4811048; doi:10.1155/2016/2596782)

Supp. 1 Virus detection reports for six data

Figure S1. HPV-18 contigs from SRR031636

Reference	Length	Read_cov(%)	#(Contig_cov%)	Depth
M20325	3135	2324(74.1)	19(62.5)	0.6(8.5)

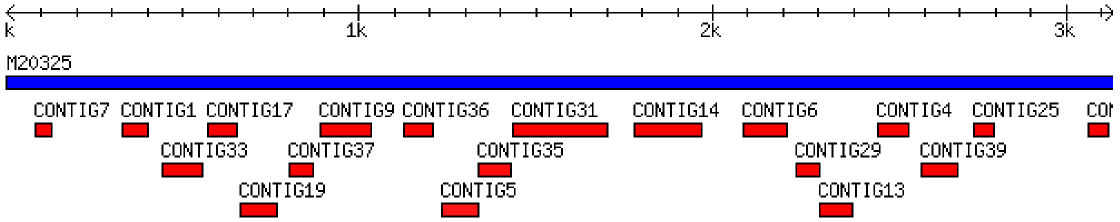

Figure S2. HBV contigs from SRR039620

Reference	Length	Read_cov(%)	#(Contig_cov%)	Depth
JQ688404	3215	1756(54.6)	7(13.3)	0.2(6.0)

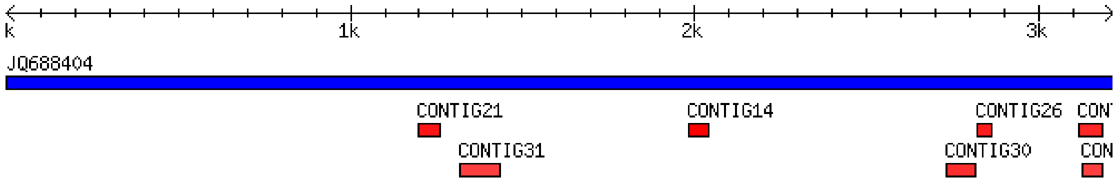

Figure S3. HCV contigs from SRR039622

Reference	Length	Read_cov(%)	#(Contig_cov%)	Depth
D85516	9535	1032(10.8)	1(0.5)	0.0(1.8)

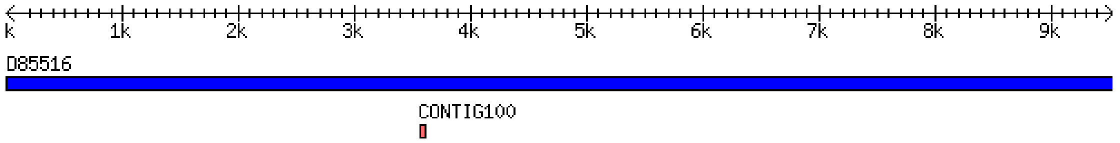

Figure S4. HIV-1 contigs from SRR941591

Reference	Length	Read_cov(%)	#(Contig_cov%)	Depth
M19921	14825	5831(39.3)	53(32.6)	3.5(210.1)

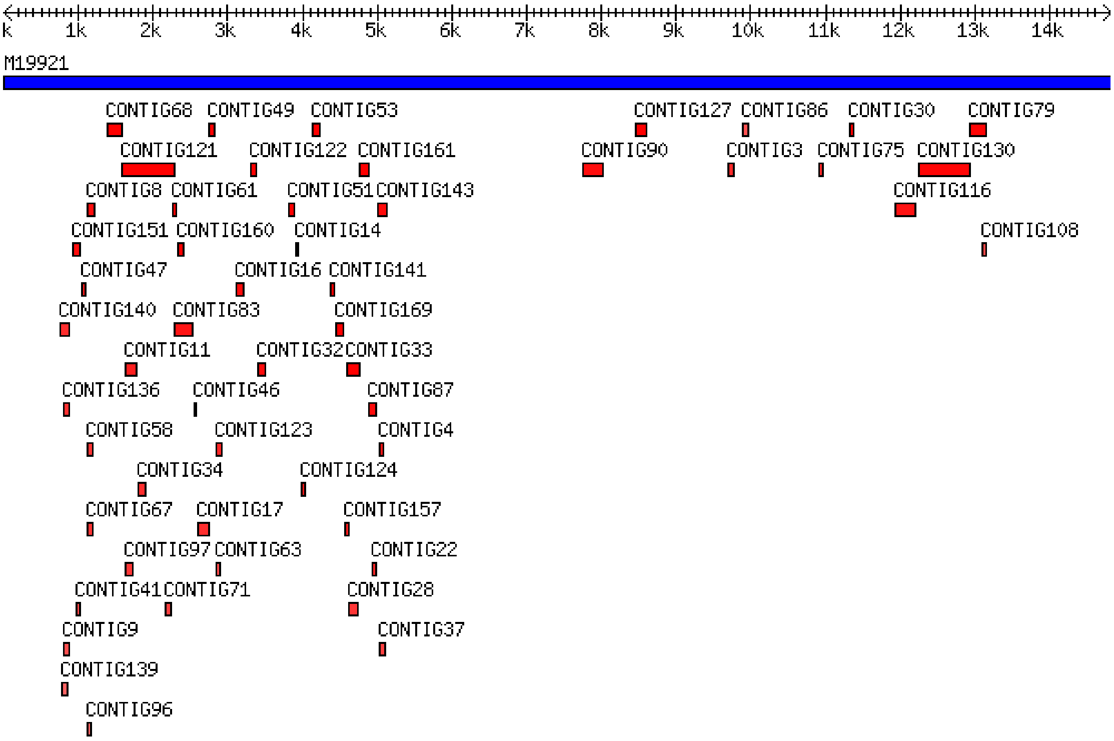

Figure S5. SMRV contigs from SRR1563017

Reference	Length	Read_cov(%)	#(Contig_cov%)	Depth
M23385	8785	8732(99.4)	1(76.9)	318.0(494.5)

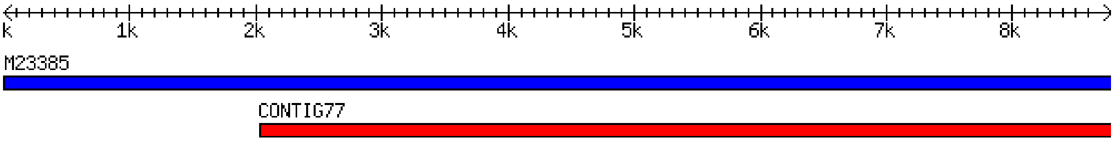

Figure S6. EBV contigs from SRR1563063

Reference	Length	Read_cov(%)	#(Contig_cov%)	Depth
M80517	184113	37898(20.6)	34(1.4)	1.2(52.8)

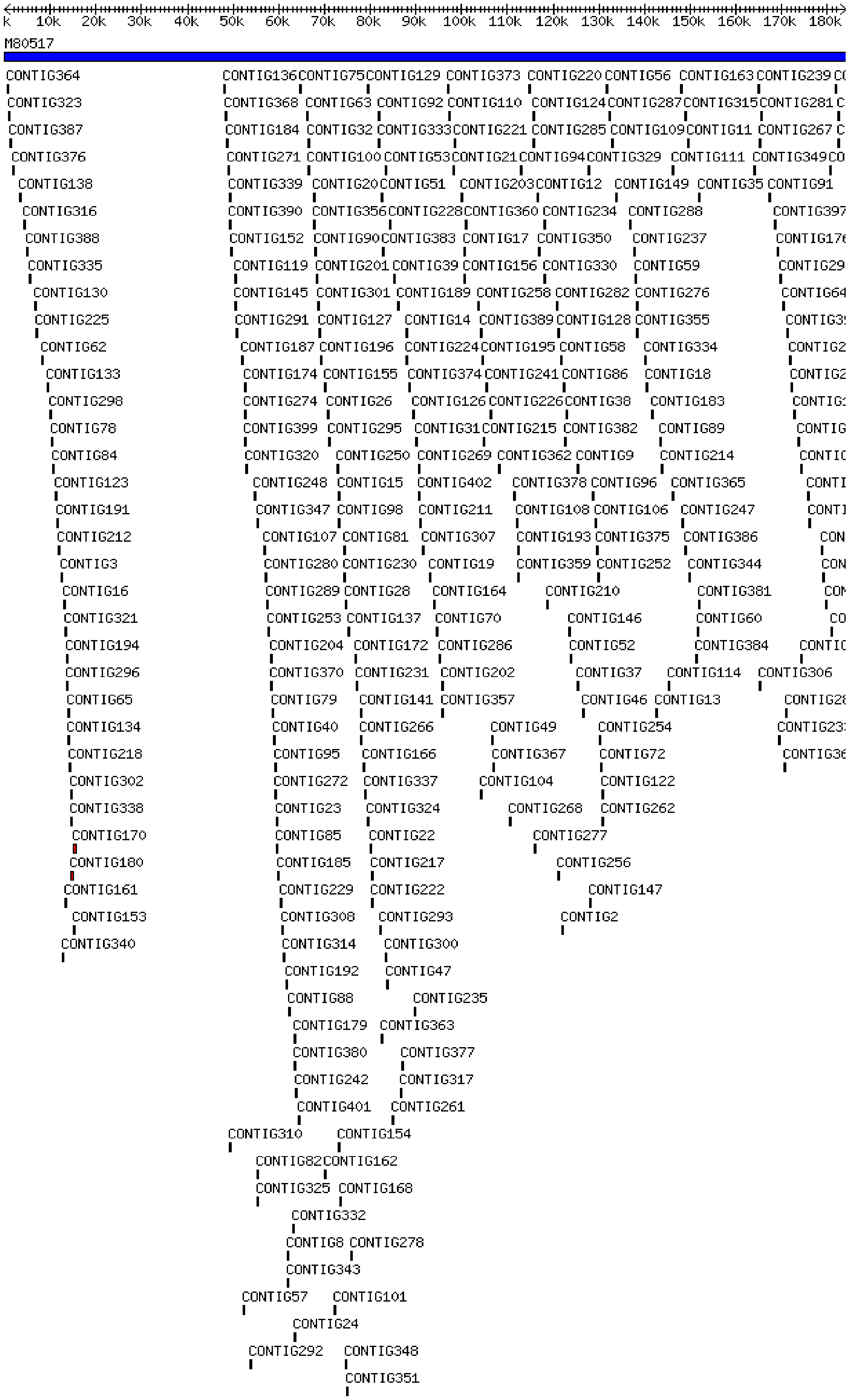

Supp. 2 Read distribution on six virus genomes

Figure S7. HPV-18 reads distribution in SRR031636

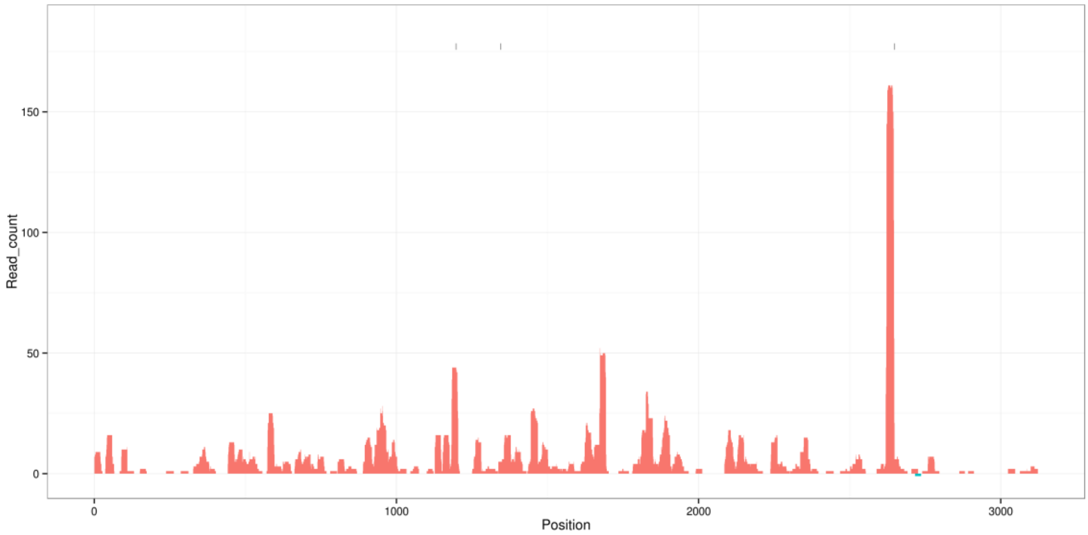

Figure S8. HBV reads distribution in SRR039620

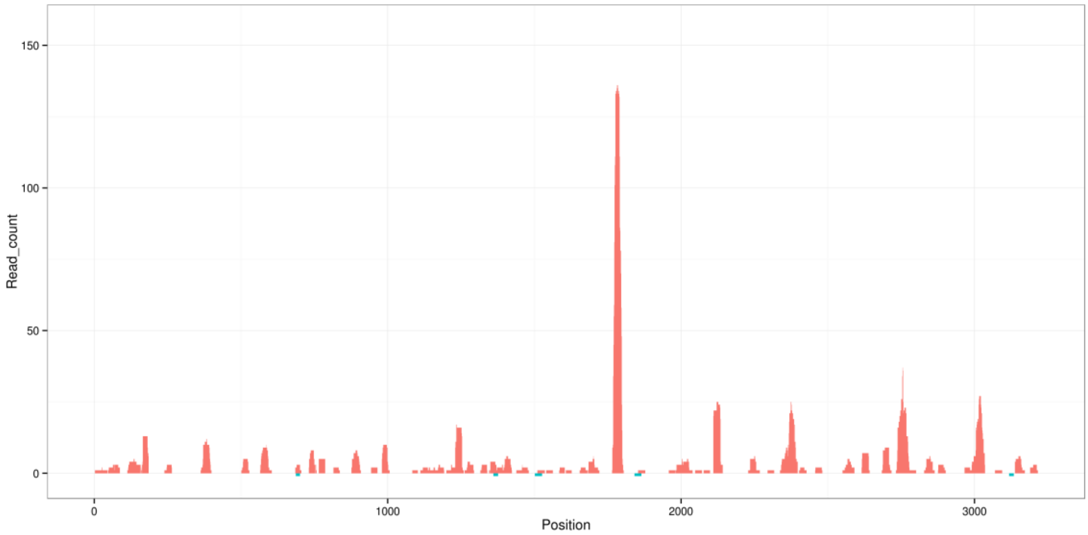

Figure S9. HCV reads distribution in SRR039622

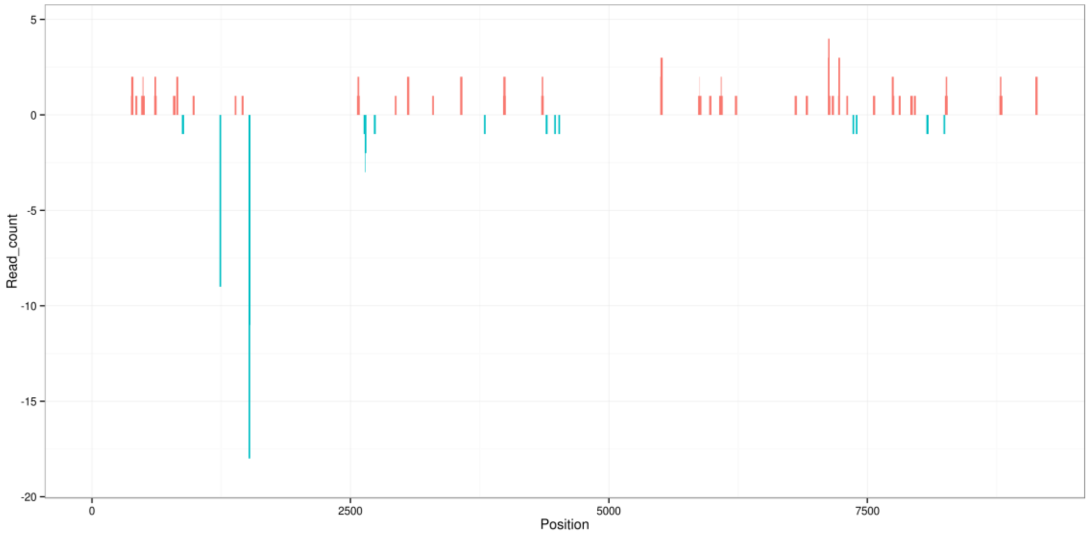

Figure S10. HIV-1 reads distribution in SRR941591

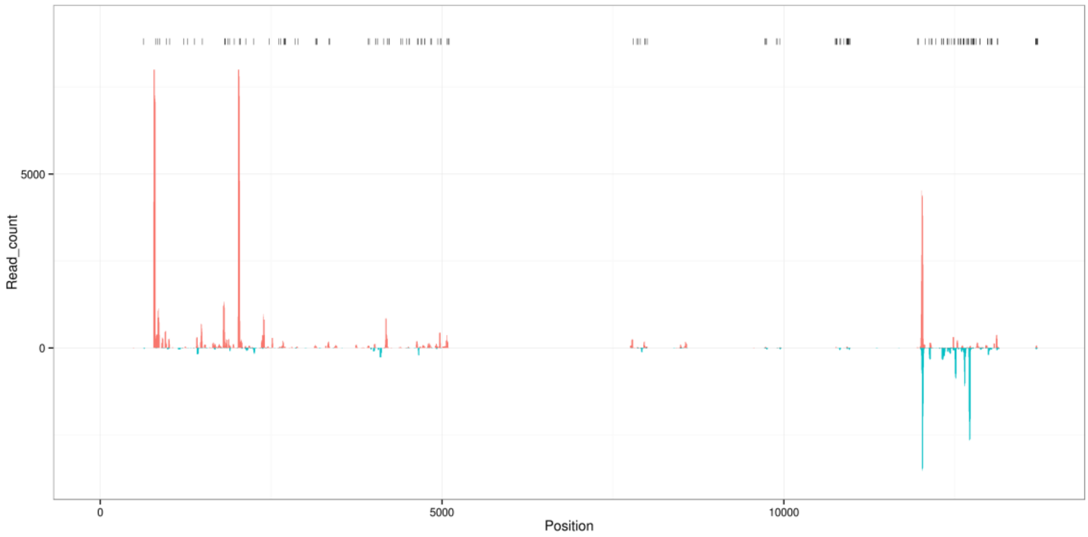

Figure S11. SMRV reads distribution in SRR1563017

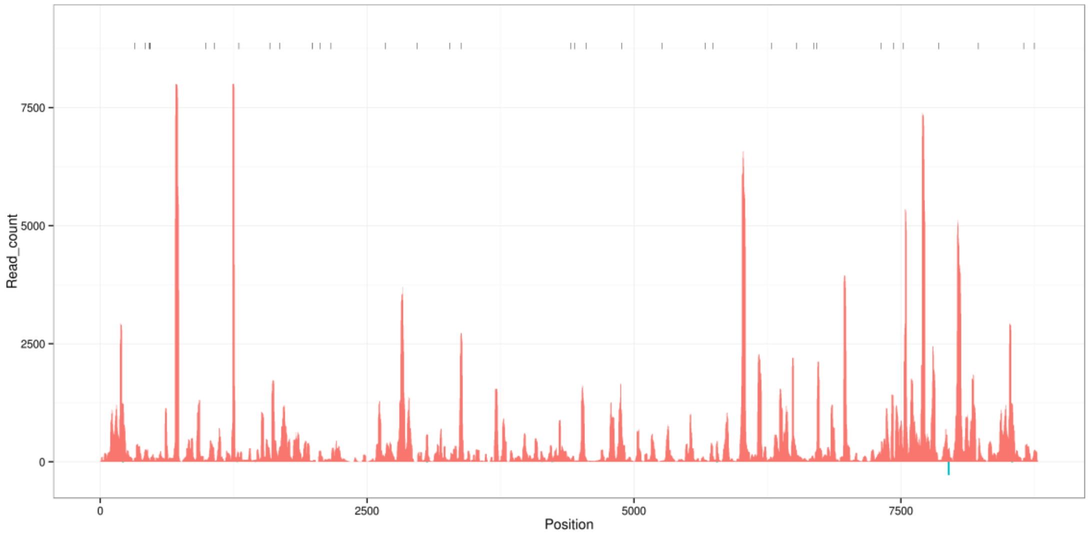

Figure S12. EBV reads distribution in SRR1563063

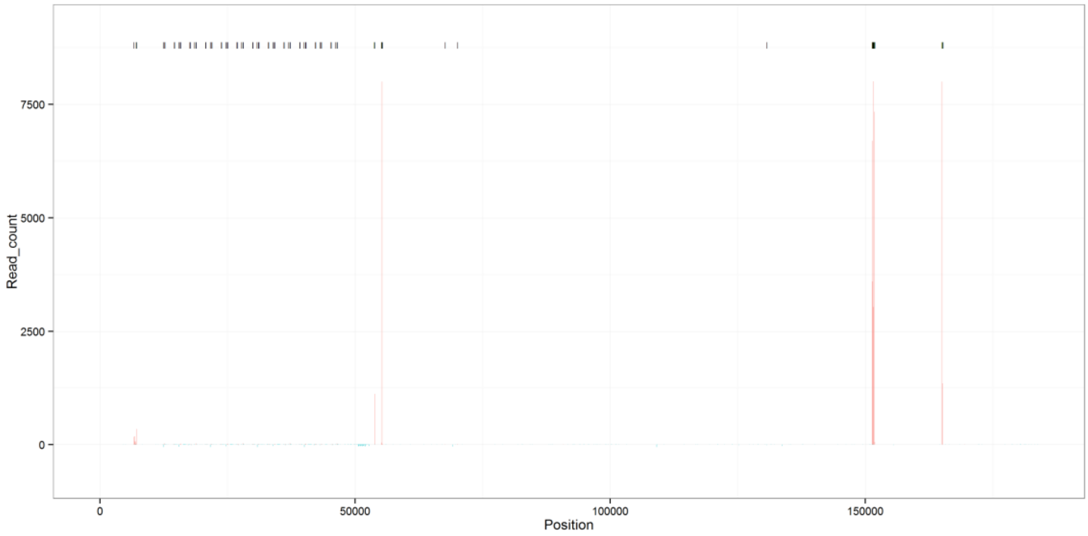

Supp. 3 Four miRNAs from seven duplexes

1. The first mature miRNA:

```
============================miRNA============================
  CCCCGGGCCTGAAGAGGTTGAC
TTGGGGCCCGGACTTCTCCAAC

Query: 1-22 rlcv-miR-rL1-1-3p: 1-22 score: 101 evalue: 0.004
UserSeq               1   ccccgggccugaagagguugac  22
                          | ||||||||||||||||||||
rlcv-miR-rL1-1-3p     1   cuccgggccugaagagguugac  22
```

2. The second mature miRNA:

```
============================miRNA============================
  TCTTAGTGGAAGTGACGTGCTG
CCAGAATCACCTTCACTGCACG
Query: 1-20 ebv-miR-BART1-5p: 1-20 score: 100 evalue: 0.004
UserSeq               1   ucuuaguggaagugacgugc  20
                          ||||||||||||||||||||
ebv-miR-BART1-5p      1   ucuuaguggaagugacgugc  20
```

3. The third mature miRNA:

```
============================miRNA============================
  TAGCCCCGCTATCCACTATGTC
GTATCGGGGCGATAGGTGATAC

Query: 1-22 ebv-miR-BART1-3p: 1-22 score: 101 evalue: 0.003
UserSeq               1   uagccccgcuauccacuauguc  22
                          |||| |||||||||||||||||
ebv-miR-BART1-3p      1   uagcaccgcuauccacuauguc  22
```

4. The predicted miRNA:

```
============================miRNA============================
GCCGGGCGGCCGCCGGTGGGTC
CGGCCCGCCGGCGGCCACCCAG
```

Predicted miRNA precursor sequence, 90 bp, dG = -69.8 [Initially -69.3]
gccgggcggccgccggtgggtcCGCTGGGCCGCTGCCCCGCTCCGGGTGGGGGTGG
CCCCGCTGGGCACCGCTGCGCCGCCGCCAGGT
15May03-09-11-36-1db38914b3 is the 2584933rd nucleic acid sequence folded on the RNA Institute mfold server.
- Sunday, May 03 09:11:38 EDT 2015 –

The repeated regions on the M80517 reference sequence.

```
>M80517:50578-50702
50578                                        50623                                                                              50702
CCGCCAGGTCCTGGGGCAGCCGGGGTTCCTGGCGCTCCGGGGGCAgccgggcggccgccggtgggtcCGCTGGGCCGCTGCCCCGCTCCGGGTGGGGGTGGCCCCGCTGGGCACCGCTGCGCCG
>M80517:50703-50827
CCGCCAGGTCCTGGGGCAGCCGGGGTTCCTGGCGCTCCGGGGGCAgccgggcggccgccggtgggtcCGCTGGGCCGCTGCCCCGCTCCGGGTGGGGGTGGCCCCGCTGGGCACCGCTGCGCCG
>M80517:50828-50952
CCGCCAGGTCCTGGGGCAGCCGGGGTTCCTGGCGCTCCGGGGGCAgccgggcggccgccggtgggtcCGCTGGGCCGCTGCCCCGCTCCGGGTGGGGGTGGCCCCGCTGGGCACCGCTGCGCCG
>M80517:50953-51077
CCGCCAGGTCCTGGGGCAGCCGGGGTTCCTGGCGCTCCGGGGGCAgccgggcggccgccggtgggtcCGCTGGGCCGCTGCCCCGCTCCGGGTGGGGGTGGCCCCGCTGGGCACCGCTGCGCCG
>M80517:51078-51202
CCGCCAGGTCCTGGGGCAGCCGGGGTTCCTGGCGCTCCGGGGGCAgccgggcggccgccggtgggtcCGCTGGGCCGCTGCCCCGCTCCGGGTGGGGGTGGCCCCGCTGGGCACCGCTGCGCCG
>M80517:51203-51327
CCGCCAGGTCCTGGGGCAGCCGGGGTTCCTGGCGCTCCGGGGGCAgccgggcggccgccggtgggtcCGCTGGGCCGCTGCCCCGCTCCGGGTGGGGGTGGCCCCGCTGGGCACCGCTGCGCCG
>M80517:51328-51452
CCGCCAGGTCCTGGGGCAGCCGGGGTTCCTGGCGCTCCGGGGGCAgccgggcggccgccggtgggtcCGCTGGGCCGCTGCCCCGCTCCGGGTGGGGGTGGCCCCGCTGGGCACCGCTGCGCCG
>M80517:51453-51577
CCGCCAGGTCCTGGGGCAGCCGGGGTTCCTGGCGCTCCGGGGGCAgccgggcggccgccggtgggtcCGCTGGGCCGCTGCCCCGCTCCGGGTGGGGGTGGCCCCGCTGGGCACCGCTGCGCCG
>M80517:51578-51702
CCGCCAGGTCCTGGGGCAGCCGGGGTTCCTGGCGCTCCGGGGGCAgccgggcggccgccggtgggtcCGCTGGGCCGCTGCCCCGCTCCGGGTGGGGGTGGCCCCGCTGGGCACCGCTGCGCCG
>M80517:51703-51827
CCGCCAGGTCCTGGGGCAGCCGGGGTTCCTGGCGCTCCGGGGGCAgccgggcggccgccggtgggtcCGCTGGGCCGCTGCCCCGCTCCGGGTGGGGGTGGCCCCGCTGGGCACCGCTGCGCCG
>M80517:51828-51952
CCGCCAGGTCCTGGGGCAGCCGGGGTTCCTGGCGCTCCGGGGGCAgccgggcggccgccggtgggtcCGCTGGGCCGCTGCCCCGCTCCGGGTGGGGGTGGCCCCGCTGGGCACCGCTGCGCCG
>M80517:51953-52077
CCGCCAGGTCCTGGGGCAGCCGGGGTTCCTGGCGCTCCGGGGGCAgccgggcggccgccggtgggtcCGCTGGGCCGCTGCCCCGCTCCGGGTGGGGGTGGCCCCGCTGGGCACCGCTGCGCCG
>M80517:52078-52115
CCGCCAGGTCCTGGGGCAGCCGGGGTTCCTGGCGCTCC
```

Supp. 4 The predicted second structures of seven duplexes

1. The first mature miRNA:

pre-miRNA	Length	C+G	MFE(-)	MFEI
rlcv-mir-rL1-1	85	49	37.70	0.91
GGCCGGCUCCUUAUUAACCUGAUCAGCCCCGGGGUUGCCUGUCU UAGCACUAA**cuccgggccugaagagguugac**AAGAAGGGUC				

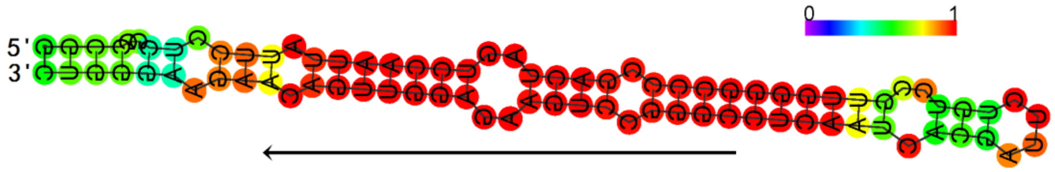

Note: the mature miRNA is in lower case letters

2. The second and third mature miRNA:

pre-miRNA	Length	C+G	MFE(-)	MFEI
ebv-mir-BART1	70	39	30.57	1.12
GGGGG**ucuuaguggaagugacgugcug**UGAAUAC AGGUCCA**uagcaccgcuauccacuauguc**UCGCCCG				

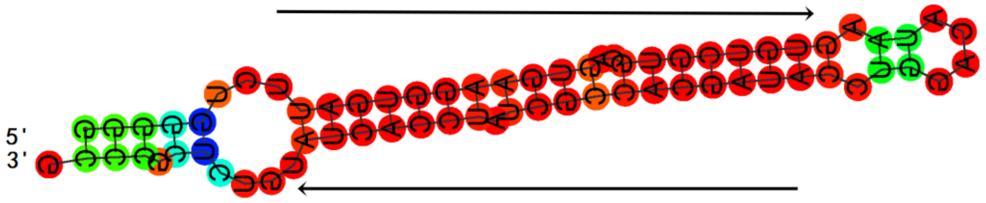

3. The predicted mature miRNA:

pre-miRNA	Length	C+G	MFE(-)	MFEI
new finding	90	77	69.46	1.00
**gccgggcggccgccggtgggtc**CGCTGGGCCGCTGCCCCGCTCC GGGTGGGGGGTGGCCCCGCTGGGCACCGCTGCGCCGCCGCCAGGT				

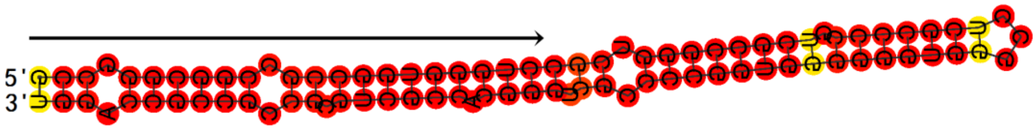

4. The unknown 1 and 2 duplexes:

Two unknown duplexes	Length	C+G	MFE(-)	MFEI
NA	76	65	46.46	0.94

CCCC**ccagacccgggtctcggccag**CCGAGCCGACCGGCCC<br>CGCGCCTGG**cgcctcctcggggccagccgccg**CCGGGG

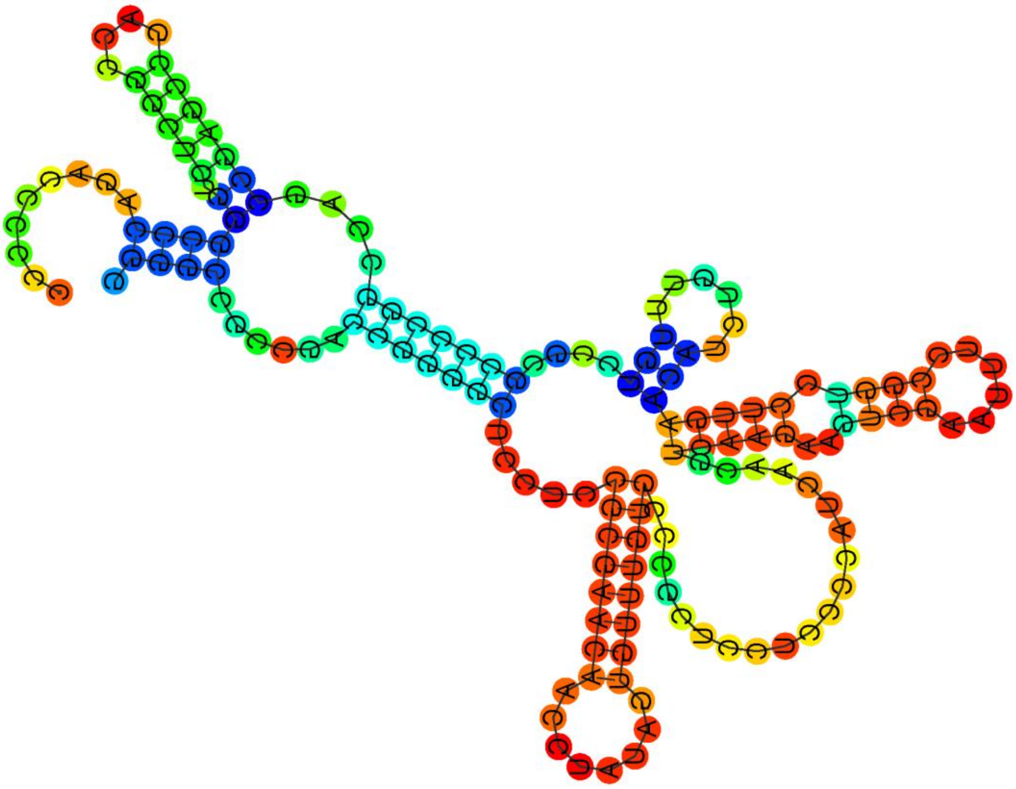

5. The unknown 3 duplex:

**unknown duplex**	**Length**	**C+G**	**MFE(-)**	**MFEI**
NA	80	39	10.4	0.33
**tttgtctacaagttcctggcc**TTTAAGCTGAAGAACTGCAACTACCCCTCCTCGCCC<br>GTGTTTGTGATATCCAACAACGG				

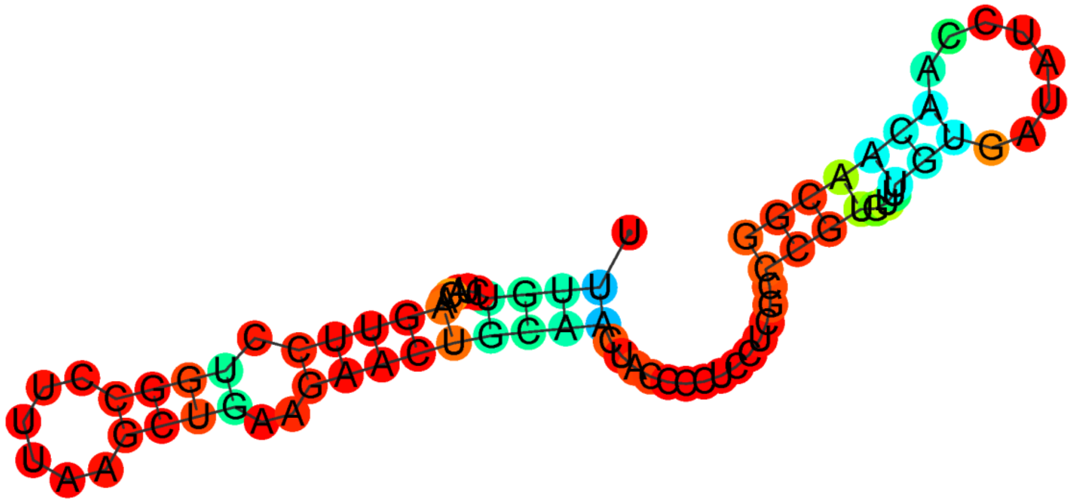

Supplement: Supplementary file 1 — Supplementary Material includes four files, which are Detection reports of six viruses, Read distribution on six virus genomes, The sequences and loci of seven duplexes, and The predicted second structures of seven duplexes. [file 2596782.f1.pdf]
